# Adherence to Methotrexate therapy in Rheumatoid Arthritis

**DOI:** 10.12669/pjms.322.9566

**Published:** 2016

**Authors:** Nasim Arshad, Nighat Mir Ahmad, Muhammad Ahmed Saeed, Saira Khan, Shabnam Batool, Sumaira Farman

**Affiliations:** 1 Dr. Nasim Arshad, FCPS. Division of Rheumatology, Fatima Memorial Hospital, Shadman. Lahore, Pakistan; 2 Prof, Nighat Mir Ahmad, MD, DABIM, DABR. Division of Rheumatology, Fatima Memorial Hospital, Shadman. Lahore, Pakistan; 3 Dr. Mohammad Ahmed Saeed, FCPS. Division of Rheumatology, Fatima Memorial Hospital, Shadman. Lahore, Pakistan; 4 Dr. Saira Khan, MRCP. Division of Rheumatology, Fatima Memorial Hospital, Shadman. Lahore, Pakistan; 5 Dr. Shabnam Batool, FCPS. Division of Rheumatology, Fatima Memorial Hospital, Shadman. Lahore, Pakistan; 6 Dr., Sumaira Farman, FRCP. Division of Rheumatology, Fatima Memorial Hospital, Shadman. Lahore, Pakistan

**Keywords:** Adherence, Methotrexate, Rheumatoid arthritis

## Abstract

**Objective:**

To determine adherence to methotrexate (MTX) therapy in patients with Rheumatoid Arthritis (RA) and to identify factors that promote either adherence or non adherence.

**Methods:**

One hundred Rheumatoid Arthritis patients on MTX for at least two months were enrolled. Questionnaire was completed by direct interview. Details recorded were, demographics (age, sex, education, monthly income), disease duration, duration on MTX and current dose. Disease Activity Score on 28 joint counts (DAS 28) at the current visit, concomitant drugs taken and number of doses of MTX missed in the previous 8 weeks were noted. Non adherence was defined as omission of any three or more prescribed doses of MTX in previous 8 week. Patients were asked for the factors that motivated their adherence to MTX as well as factors for non adherence. Presence of side effects due to MTX was also recorded.

**Result:**

Non adherence was found among 23% of cases. Patients of low socioeconomic group (p <0.0001) and on MTX for longer duration (p <0.001) had higher non adherence. Non adherent patients had significantly higher disease activity as measured by DAS 28 (p<0.001). Good counseling and education by the doctor was a strong predictor of adherence (p <0.001). Lack of affordability (p <0.001); lack of availability at local pharmacy (p <0.001); lack of family support (p <0.001) and lack of awareness regarding need and importance of MTX (p < 0.001were found as significant factors for non adherence.

**Conclusion:**

MTX non adherence in RA is noted in about one fourth of study group. Various economical and social issues lead to non adherence but good patient education and counseling by doctor could promote adherence in this study group.

## INTRODUCTION

Rheumatoid arthritis (RA) is a chronic systemic auto immune inflammatory disease with a prevalence of approximately 1%.^1^, ^2^ If untreated, RA leads to severe progressive joint damage, functional disability, morbidity and increase mortality. Insight into the molecular pathogenesis of RA has lead to an increasing number of targeted diseases modifying anti rheumatoid drugs (DMARDs).^3^, ^4^ Early intervention with these DMARDs prevents joint damage and improves long term functional outcome.^5^, ^6^ In this era a realistic goal for every patient with RA is low disease activity or disease remission.^7^

Methotrexate (MTX) is the most widely used DMARD in the treatment of RA. Well designed long term studies have proved MTX to be both more efficacious and better tolerated than other DMARDs.^6^, ^8^ According to ACR and EULAR treatment guidelines for RA, MTX is an anchor drug either alone or in combination with conventional or biologic DMARDs.^8^

MTX is also extensively used for other auto immune conditions like psoriatiric arthritis, juvenile idiopathic arthritis, uveitis, vasculitis, inflammatory bowel disease. As in all chronic diseases where compliance to therapy is vital for success of treatment, adherence to MTX is the key to attaining the goal of disease remission or low disease activity.^9^ Non adherence to treatment may jeopardize patient’s health, cause unnecessary clinic appointments and diagnostic studies as well as additional treatments. These effects increase the cost of medical care and can contribute to increased morbidity and mortality.^10^, ^11^ The estimated cost of non adherence to standard treatment in chronic diseases in the US is US hundred billion dollars annually.^10^

The purpose of this study was to determine adherence to MTX therapy in our patients with RA and to look for factors that may positively or adversely influence adherence. This would help guide rheumatologists to modify these factors, thereby increasing adherence to this important anchor drug.

## METHODS

This was a cross sectional observational study conducted at Fatima Memorial Hospital, Division of Rheumatology out patient’s clinic over a period of three months from May 2015 to July 2015. Institutional review board of Fatima Memorial Hospital approved the study. A total of hundred patients diagnosed with RA as per American College of Rheumatology 1987 criteria were included in this study. All patients had been on MTX for more than two months. After informed consent a pre-tested questionnaire was completed by the researcher.

Demographic data like age, sex, address were recorded. In this study education level was defined as; no formal education, less than or equal to matriculation or more than matriculation. Socioeconomic status was ascertained by using in arbitrary monthly income figure of up to rupees 35,000 or more than rupees 35,000.^12^ Duration of symptoms compatible with RA in years, duration on Methotrexate therapy in months, current dose of MTX in mg / week were recorded. Disease activity on the current visit was calculated by disease activity score (DAS) 28 which has four variables; tender joint count, swollen joint count, patient pain visual analogue scale (VAS) and erythrocyte sedimentation rate (ESR). Concomitant DMARDs, non-steroidal anti-inflammatory drugs (NSAIDS) and steroids taken by patient were noted.

Adherence was defined as omission of two or less doses of prescribed Methotrexate during previous 8 weeks. This number was used because two times or less would represent adherence rate of 80% or more which is considered acceptable by most authors.^11^, ^13^, ^14^ Patients who missed any three or more doses were considered non adherent.

Reasons for non adherence were explored. Several factors were identified including forgetfulness; lack of affordability; lack of availability at local pharmacy; social myths like fear of side effects, other people’s negative advice, fear of dependency; lack of awareness regarding importance and long term need of drug; lack of social support when patient needed family support to get the medicine which was not available. All patients were also asked for the factors that encouraged them to take treatment. These factors included pain relief; improved quality of life, fear of disability, education and counseling by doctors and trust on doctors. The patients were asked for occurrence of side effects attributable to MTX like nausea, vomiting, fatigue, anorexia, headache, recurrent oral ulcer, pain epigastrium or others.

### Statistical analysis

For population characteristics Mean ± standard deviation was calculated to summarize the quantitative variables; N (%) was calculated to summarize qualitative data. For the comparison of quantitative variables between adherent and non adherent groups, independent sample t-test was applied to see the difference between means and standard deviations of both groups. For the comparison of qualitative variables between adherent and non adherent groups Chi-square test was applied to compare within group differences.

## RESULTS

The characteristics of the study population are shown in Table-I. Mean age was 41.5 ± 11.2 years. Females were 73%. Over all non adherence rate was 23%. Age, gender, level of education, duration of RA, current dose of MTX, concomitant use of other DMARDs, NSAIDs and steroids was not significantly different between adherent and non adherent patients. However patients who fulfilled the criteria for non adherence had increased disease activity score DAS 28 (p value < 0.001), had been on MTX for longer periods (p value < 0.001) and belonged to low socioeconomic group (p value <0.0001). Side effects profile was not significantly different between the adherent and non adherent patients (Table-II). Comparison of motivation factors for adherence between the adherent and non adherent groups is shown in Table-III. Good education and counseling by doctor was a strong motivation factor with p value <0.001. Lack of affordability, lack of availability at local pharmacy, lack of awareness regarding importance of long term need of drug, lack of family support were statistically significant factors(p value <0.001) for non adherence (Table-IV).

**Table-I:** Population Characteristics.

	Total (n= 100)	Adherent (n= 77)	Non-Adherent (n= 23)	p-value
Age	41.55 ± 11.24	41.82 ± 11.68	40.65 ± 9.81	0.665
Gender				p-value
Female	73 (73%)	54(74.0%)	19(26.0%)	0.237
Education				p-value
Matric or below	44 (44%)	35(79.5%)	9(20.5%)	0.072
More than matric	26(26%)	23(88.5%)	3(11.5%)	
None	30(30%)	19(63.3%)	11(36.7%)	
Socio Economic Status				p-value
<=35,000/month	58 (58%)	36(62.1%)	22(37.9%)	< 0.001
>35,000/month	42 (42%)	41(97.6%)	1(2.4%)	
Duration of disease in years	6.24 ± 3.96	6.10 ±4.09	6.70±3.55	0.530
Current dose of methotrexate (mg/week)	18.58±5.13	18.15±5.02	20.00±5.33	0.130
DAS 28 Score	4.59 ±1.13	4.38±1.10	5.32±0.92	< 0.001
Duration of treatment with methotrexate (months)	48.44±36.29	45.71±35.84	57.57± 37.08	< 0.001
Taking Steroid	52 (52%)	36(69.2%)	16(30.8%)	0.055
Taking NSAIDS	67 (67%)	48(71.6%)	19(28.4%)	0.070
Taking Concomitant DMARDS	94 (94%)	71(75.5%)	23(24.5%)	0.167

**Table-II:** Side effect Profile.

	Total (n= 100)	Adherent (n= 77)	Non-Adherent (n= 23)	p-value
Nausea	66(66.0%)	50(75.8%)	16(24.2%)	0.681
Vomiting	14(14.0%)	11(78.6%)	3(21.4%)	0.880
Fatigue	23(23.0%)	14(60.9%)	9(39.1%)	0.036
Anorexia	20(20.0%)	15(75.0%)	5(25.0%)	0.812
Headache	11(11.0%)	9(81.8%)	2(18.2%)	0.687
Recurrent oral ulcer	26(26.0%)	20(76.9%)	6(23.1%)	0.991
Pain epigastrium	28(28.0%)	25(89.3%)	3(10.7%)	0.069

**Table-III:** Factors affecting adherence.

	Total (n= 100)	Adherent (n= 77)	Non-Adherent (n= 23)	p-value
Pain relief	80(80.0%)	59(73.8%)	21(26.3%)	.122
Improve quality of life	33(33.0%)	26(78.8%)	7(21.2%)	.766
Fear of disability	9(9.0%)	7(77.8%)	2(22.2%)	.954
Good counseling by doctors	78(78.0%)	72(92.3%)	6(7.7%)	<0.001*
Faith on doctors	40(40.0%)	35(87.5%)	5(12.5%)	.042*

**Table-IV:** Reasons of Non-adherence.

	Total (n= 100)	Adherent (n= 77)	Non-Adherent (n= 23)	p-value
Forgetfulness	68(68.0%)	51(75.0%)	17(25.0%)	.488
Affordability	18(18.0%)	2(11.1%)	16(88.9%)	<0.001*
Availability	11(11.0%)	1(9.1%)	10(90.9%)	<0.001*
Social Myths	0(0.0%)	0(0.0%)	0(0.0%)	(NA)
Lack of awareness regarding importance and long term need of drug	17(17.0%)	2(11.8%)	15(88.2%)	<0.001*
Lack Of Social Support	13(13.0%)	0(.0%)	13(100.0%)	<0.001*

## DISCUSSION

We found that 77% of patients prescribed MTX for RA were adherent to the medications. Although we do not have local or regional studies to show exact figures for adherence to MTX in our RA patients a study conducted in Lahore in 2001 showed that adherence to MTX can be improved by folic acid supplementation which is now the standard of care.^15^ Indian studies have showed that MTX monotherapy had higher retention rate and adherence when compared with other DMARDs for treatment of RA.^16^, ^17^ International studies for adherence to MTX show results comparable to ours. One large American Cohort study including more than 14,000 patients^18^ with RA and prevalence study including 2662 RA patients^19^ reported compliance rate of 65-80%. A ten year longitudinal study conducted at Denmark including 941 patients with RA showed adherence rate of 80%.^20^

In our study age, gender, education, concomitant drug use, dose of MTX was not found to be associated with non compliance. Non adherent patients had more active disease as shown by statistically significant increase DAS 28 score. This is not unexpected result as non adherence is associated with poorly controlled disease as shown in other studies.^9^, ^21^-^23^

In our study patients of low socioeconomic class were more likely to be non adherent. About 70% of non adherent patients reported affordability issue leading to non compliance. Similar results have been reported by a study simultaneously conducted for MTX adherence in JIA in USA and Brazil, where patients from Brazil were more likely to be non adherent due to affordability issue.^14^ In addition they also reported lack of availability of MTX at local pharmacy which was also statistically significant factor contributing to non adherence to our study (P value < 0.001). Higher ‘out of pocket costs’ have also been associated with non adherence in western countries.^23^, ^24^ These findings highlight economics importance in our patients. Despite the fact that this study was conducted in a setup where DMARDs are provided free of cost to patients who sign up for financial support, they had problems reaching the hospital in time to get medical refills as they could not afford intracity travel fares. This problem was manifolds for patients from other cities who also reported lack of availability of MTX at local pharmacy. Similarly lack of family support led to non adherence in 56% of non adherent patients in contrast to good social help reported by 100% of adherent patients. Social issues were specially faced by female patients who depend on their male relative for medicine refills due to cultural norms in our society.

Interestingly none of the participants of the study reported missing the drug dose due to fear of side effects, other people’s negative advice, fear of dependency on this drug. This may be as this study was conducted in a tertiary care outpatient rheumatology clinic which indicates their faith in allopathic system of medicine and their level of awareness but this need to be further studied.

Lack of awareness regarding importance of MTX as an anchor drug for treatment of RA and its long term need was reported by 65% of non adherent patients. This in addition to the finding that good patient education and counseling by the doctors regarding importance and need of MTX is a strong predictor for adherence highlights the importance of patient education. Enormous body of research data supports educating the patients about their illness, need and importance of treatment, any potential side effects and coping strategies.^25^, ^26^

### Limitations of the study

We relied on patient self reports to determine adherence which is subject to recall and social desirability bias. This study has been conducted in a tertiary care rheumatology clinic where rheumatologists greatly emphasize on patient education, so further long term population based studies are needed to generalize the result.

In conclusion about one fourth of our study patients were non adherent to MTX. This is an alarmingly high proportion as improving medication adherence with available DMARDs would improve treatment effectiveness and health care costs. An improved doctor patient communication as well as better health care delivery and social support systems are very much the need of today to maintain optimal standards of healthcare.
